# Comparison of waveform-derived corneal stiffness and stress-strain extensometry-derived corneal stiffness using different cross-linking irradiances: an experimental study with air-puff applanation of ex vivo porcine eyes

**DOI:** 10.1007/s00417-020-04792-8

**Published:** 2020-06-17

**Authors:** Robert Herber, Mathew Francis, Eberhard Spoerl, Lutz E. Pillunat, Frederik Raiskup, Abhijit Sinha Roy

**Affiliations:** 1 Department of Ophthalmology, University Hospital Carl Gustav Carus, TU Dresden, Fetscherstraße 74, 01307 Dresden, Germany; 2 Narayana Nethralaya Foundation, Bangalore, India

**Keywords:** Biomechanics, Cornea, Corvis ST, Dynamic Scheimpflug analyzer, Porcine, Stress-strain measurement, Strip extensometry

## Abstract

**Purpose:**

To assess corneal stiffening of standard (S-CXL) and accelerated (A-CXL) cross-linking protocols by dynamic corneal response parameters and corneal bending stiffness (Kc[mean/linear]) derived from Corvis (CVS) Scheimpflug-based tonometry. These investigations were validated by corneal tensile stiffness (K[ts]), derived from stress-strain extensometry in ex vivo porcine eyes.

**Methods:**

Seventy-two fresh-enucleated and de-epithelized porcine eyes were soaked in 0.1% riboflavin solution including 10% dextran for 10 min. The eyes were separated into four groups: controls (*n* = 18), S-CXL (intensity in mW/cm^2^*time in min; 3*30) (*n* = 18), A-CXL (9*10) (*n* = 18), and A-CXL (18*5) (*n* = 18), respectively. CXL was performed using CCL Vario. CVS measurements were performed on all eyes. Subsequently, corneal strips were extracted by a double-bladed scalpel and used for stress-strain measurements. K[ts] was calculated from a force-displacement curve. Mean corneal stiffness (Kc[mean]) and constant corneal stiffness (Kc[linear]) were calculated from raw CVS data.

**Results:**

In CVS, biomechanical effects of cross-linking were shown to have a significantly decreased deflection amplitude as well as integrated radius, an increased IOP, and SP A1 (*P* < 0.05). Kc[mean]/Kc[linear] were significantly increased after CXL (*P* < 0.05). In the range from 2 to 6% strain, K[ts] was significantly higher in S-CXL (3*30) compared to A-CXL (9*10), A-CXL (18*5), and controls (*P* < 0.05). At 8% to 10% strain, all protocols induced a higher stiffness than controls (*P* < 0.05).

**Conclusion:**

Several CVS parameters and Kc[mean] as well as Kc[linear] verify corneal stiffening effect after CXL on porcine eyes. S-CXL seems to have a higher tendency of stiffening than A-CXL protocols have, which was demonstrated by Scheimpflug-based tonometry and stress-strain extensometry.

## Introduction

Corneal cross-linking (CXL) was first described by Spoerl and Seiler in 1998 aiming to increase mechanical stiffness of corneal tissue [1]. Several techniques with photosensitizers (e.g., riboflavin), chemical solutions (e.g., glutaraldehyde), and aldehyde sugars (e.g., glucose) were tested for their suitability to increase stiffness primarily in the case of keratectasia [2]. Following these basic investigations, the first clinical study was published by Wollensak et al., who used the procedure of cross-linking with riboflavin and ultraviolet A (UVA) to halt the progression in keratoconic eyes [3]. Keratoconus is described as an ectatic disorder of the cornea [4], which is based on focal biomechanical weakening [5] followed by protrusion of the anterior and posterior curvature, corneal thinning, irregular astigmatism, and loss of visual acuity [6].

The most common treatment, based on early investigations of CXL, is the Dresden protocol (standard CXL, S-CXL) where an irradiance of 3 mW/cm^2^ is applied for 30 min on the treated eye after riboflavin is instilled [3]. This is equal to an energy dose of 5.4 J/cm^2^ [3] and offers a safety zone to avoid endothelium or retinal damage if the corneal thickness is more than 400 μm [7]. According to the Bunsen-Roscoe Law, the photobiological process is reciprocal and leads to a possible change of irradiance with a simultaneous adjustment of the time [8]. Therefore, several CXL protocols were tested with a shorter duration of irradiance to improve the patient’s comfort [8] and were introduced into clinical practice as accelerated CXL (A-CXL). Recently, a study was published that compared the clinical outcome after 12 months, in different protocols using topography and tomography of Scheimpflug imaging. All applied protocols showed improvement and stability concerning maximal keratometry, which is currently the most important parameter to verify the efficacy of CXL in vivo [9]. Additionally, S-CXL was more pronounced in improving corneal surface indices provided by Scheimpflug imaging [9].

The evidence of increased stiffness by CXL has so far been shown ex vivo, under laboratory conditions. Stress-strain measurements can be performed as uniaxial tensile tests using an extensometer and have been applied to measure the stiffness and/or Young’s modulus in corneal strips of porcine or human donor tissue [2, 8, 10, 11]. Another technique to determine the biomechanical properties is the inflation test, where the shape of the cornea is investigated while increasing the intraocular pressure (IOP) [12]. It could be shown that Young’s modulus was increased by factor 1.6 in inflation tests, which is comparable to stress-strain measurements in porcine eyes (factor 1.8) [10, 12]. However, these techniques are not applicable in vivo.

In vivo, attempts were made to derive the biomechanical properties of the cornea through special non-contact tonometry methods. There are two commercial devices available, the ocular response analyzer (ORA; Reichert Ophthalmic Instruments, Depew, NY, USA) and the Corvis ST (CVS; Oculus, Wetzlar, Germany). The ORA is a non-contact tonometer that detects corneal deformation after an induced air puff is applied to the cornea. The main ORA parameters corneal hysteresis (CH) and corneal resistance factor (CRF) show lower values in keratoconic eyes compared to healthy ones [13]. However, the performance of discrimination is low for these parameters in keratoconus with normal corneal thickness [13], mild keratoconus [14], and forme fruste keratoconus [15] compared to normal eyes. Furthermore, there could not be found changes after CXL in CH and CRF [16–18], as they do not describe material stiffness or in others words elasticity [18]. CH is a parameter that takes into account viscosity and elasticity [18]; thus, changes induced by CXL in elasticity might be masked by changes in viscosity [19]. A Scheimpflug-based tonometer, on the other hand, is able to detect more information during the deformation process of the cornea. The Corvis ST is a Scheimpflug analyzer that captures the deformation process of the cornea with an ultra-high-speed camera after applying an air puff [20, 21]. In the meantime, the evaluation of Corvis ST measurements has been upgraded by the development of indices that are able to distinguish between healthy and keratoconic eyes [22, 23]. The main index, called the *Corvis Biomechanical Index* (CBI), is described as a combination of certain dynamic corneal response parameters (DCR) with high sensitivity and specificity in detecting keratoconus [22]. Recently, our group has shown that CBI has greater ability in differentiating between keratoconus and healthy eyes than CH or CRF [24]. Further, the combination of topographical and tomographical data with biomechanical parameters (*Tomographic and Biomechanical Index*, TBI) is possible and enables a screening for ectasia before refractive surgery is performed [25]. Additionally, alterations in certain DCR parameters suggesting changes in biomechanical properties after CXL in keratoconic eyes have been shown in preliminary [26] and long-term results [27].

Roy and co-workers have introduced a new analytical model that derives corneal and extra-corneal stiffness from the whole deformation process recorded by Corvis ST [28, 29]. Thus, the model takes into account, non-linear elastic corneal properties as well as extra-corneal properties, e.g., eye globe, fat tissue, and muscles [28, 29]. The model has already been applied to healthy patients to investigate the impact of myopia and age [30] and to differentiate between healthy, suspect, and keratoconic eyes [29].

The aim of the current study was to assess corneal stiffening effects of standard cross-linking (S-CXL) and accelerated cross-linking (A-CXL) protocols with the help of basic parameters of the Corvis ST as well as the novel corneal stiffness parameters derived from the deformation process. These parameters describe the bending properties of the cornea caused by the perpendicular indentation from the air puff and were compared to uniaxial tensile properties of stress-strain measurements.

## Methods

### Preparation and cross-linking procedure

Porcine eyes were transported from a slaughterhouse under cool conditions to the clinical laboratory of the Department of Ophthalmology, University Hospital Carl Gustav Carus, TU Dresden, Germany. Seventy-two porcine eye globes were cleaned off the extra-ocular tissue and were separated into four different groups: 1—control group (*n* = 18 eyes); 2—standard CXL group (*n* = 18 eyes), 3 mW/cm^2^ for 30 min, S-CXL (3*30); 3—accelerated CXL group (*n* = 18 eyes), 9 mW/cm^2^ for 10 min, A-CXL (9*10); and 4—accelerated CXL group (*n* = 18 eyes), 18 mW/cm^2^ for 5 min, A-CXL (18*5). Subsequently, all eye globes were de-epithelialized using a hockey knife and afterwards soaked in 0.1% riboflavin solution including 10% dextran for 10 min. De-epithelialization was done according the “epi-off” CXL procedure that was described by Wollensak et al. [3]. The control group was only used for comparison between the CXL protocols in stress-strain measurements. However, they were also de-epithelialized and soaked in riboflavin solution to avoid different hydration conditions of the corneas.

**Fig. 1:**
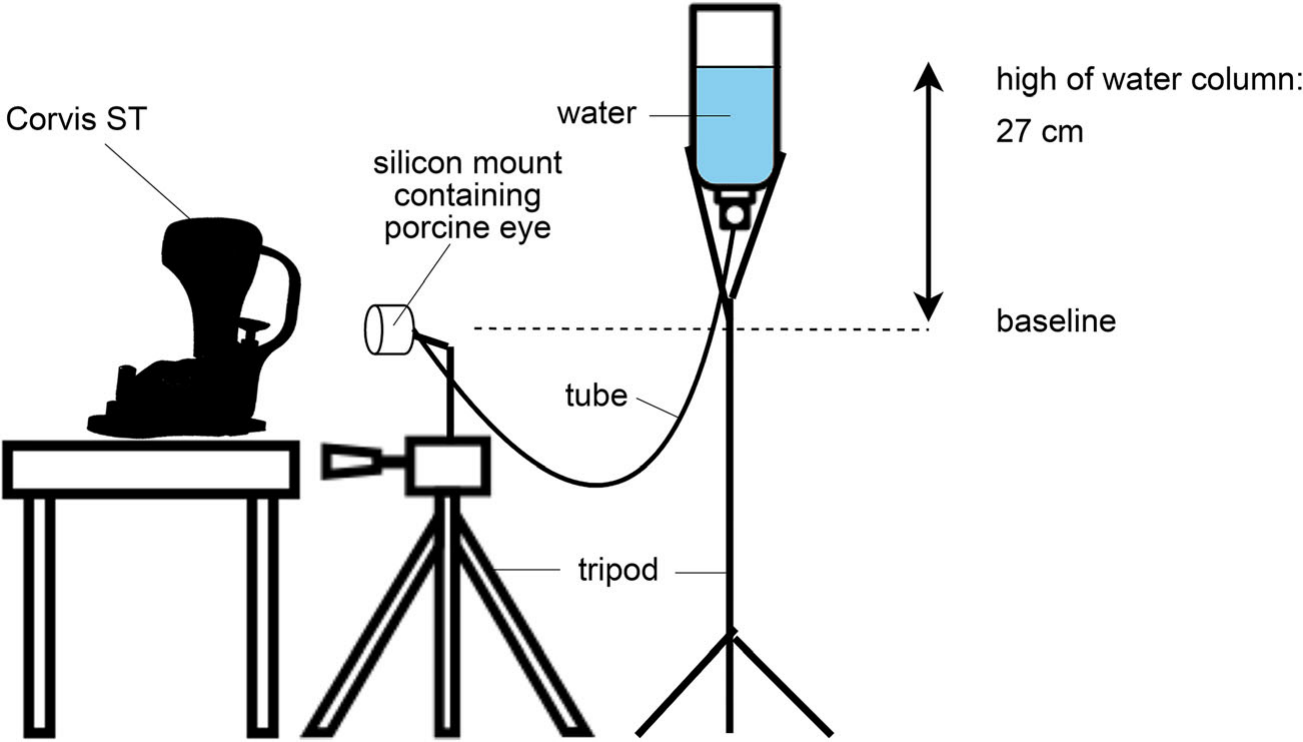
Experimental set-up of Corvis ST and manometer to ensure constant IOP conditions of 20 mmHg

**Fig. 2:**
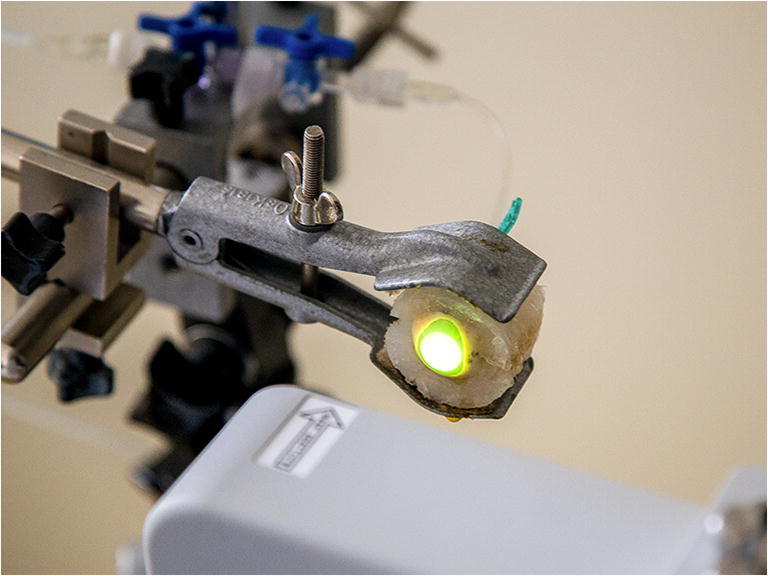
CXL device (CCL Vario) in front of the porcine eye globe mounted in a silicone case

**Fig. 3:**
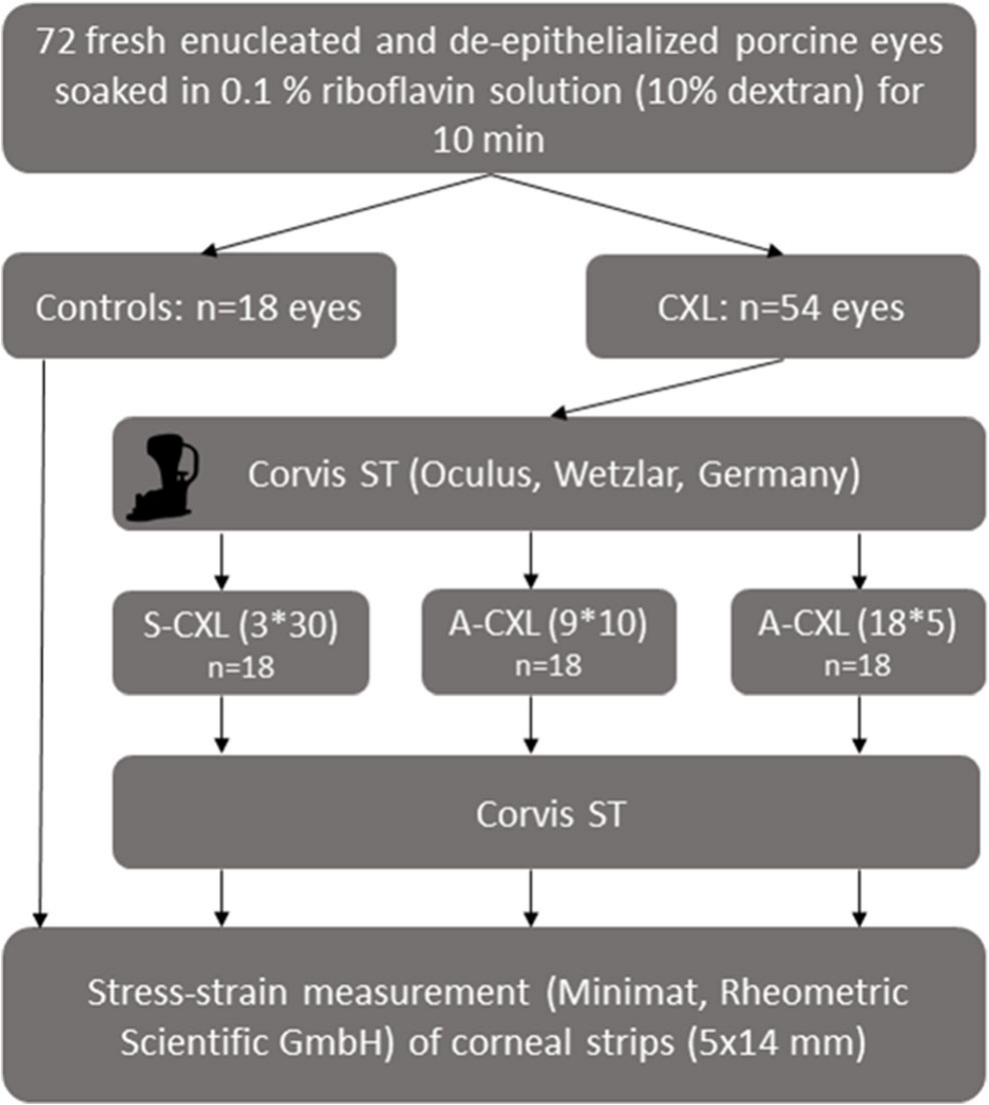
Flowchart of experimental workflow

For air-puff tonometry, the eye globes were mounted in a specially manufactured silicon case and adjusted in front of the Corvis ST. The superior-inferior meridian of the cornea was aligned to the 2-dimensional image of the Corvis ST, which was consistent to subsequent stress-strain measurement. The measurement was performed twice on each eye while they were plugged by a manometer, which was calibrated using a miniature pressure probe (Keller AG, Winterthur, Switzerland). In Fig. 1, the experimental set-up is drawn schematically. The manometer consisted of a reversed bottle containing water and was connected to the eye globe by a tube. A difference between the water surface within the bottle and the mounted eye amounted 27 cm, which ensured a constant intraocular pressure of 20 mmHg during the whole procedure. Following this, the CXL procedure was performed on groups 2, 3, and 4 using the CXL system (CCL Vario, Peschke Trade GmbH, Huenenberg, Switzerland; Fig. 2). The corneas were irradiated with a diameter of 11 mm and different intensities of irradiance (3 mW/cm^2^, 9 mW/cm^2^, and 18 mW/cm^2^). Each eye of the CXL group was measured before (pre-treatment) and after CXL (post-treatment) using the Corvis ST. The corneal surface was wetted using a water-soaked sponge releasing the air puff automatically. The complete workflow is shown in Fig. 3.

### Stress-strain measurement

**Fig. 4:**
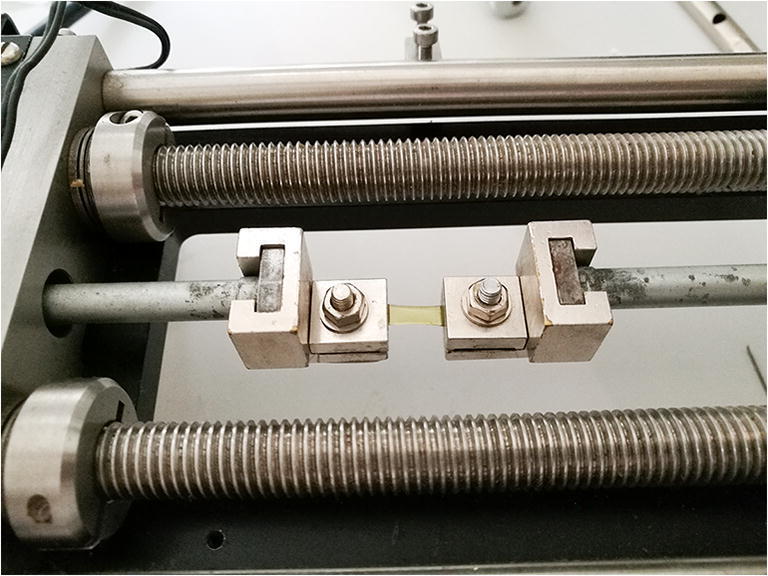
Strip of corneal tissue mounted by clamps of the extensometer during stress-strain measurement

The stress-strain measurement was performed on all groups using the microcomputer-controlled material tester (MINIMAT, Rheometric Scientific GmbH, Bensheim, Germany). Corneal strips (5 × 14 mm) were extracted by a double-bladed scalpel in superior-inferior direction and used for stress-strain measurements. After that, the corneal strips were clamped into the device and preloaded (0.02 N/m^2^) with a length of around 7 mm (Fig. 4). Each corneal strip was elongated by increasing the load from 0.02 to 3 N with a velocity of 2 mm/min. First, tensile stiffness (K[ts]) was calculated from the slope of a load/force to an extension plot by the material tester software. Second, the slope of stress-strain was exported to a spreadsheet file.

### Measurement of dynamic corneal response parameters and waveform-derived corneal stiffness parameters

**Fig. 5:**
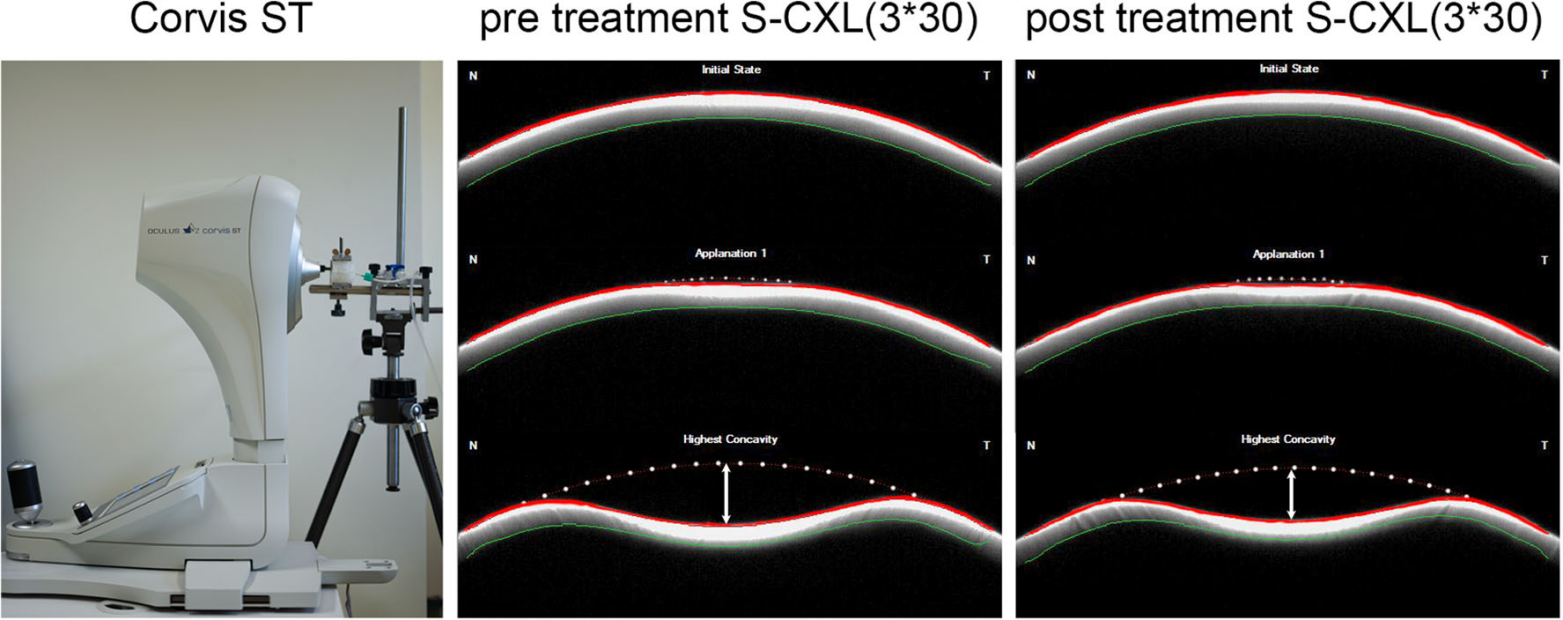
Corvis ST device in laboratory environment (left) and Scheimpflug images from pre-treatment (center) and post-treatment measurement (right) under constant IOP conditions of 20 mmHg. Dotted line represents initial anterior corneal shape. Line with arrows represents deflection amplitude

The Corvis ST (Corneal Visualization Scheimpflug Technology; Fig. 5) is known to be a dynamic Scheimpflug analyzer that records the corneal deformation process with an ultra-high-speed camera (4300 frames per second). The cornea is forced from its initial convex shape into a first applanation state (A1), followed by a concave phase until the highest concavity (HC) is reached and recovery to the initial state occurs (Fig. 5, center), passing the second applanation state (A2) [20]. The observed corneal diameter accounts for 8 mm. Different dynamic corneal response (DCR) parameters were released during device development. There are basic parameters, e.g., deformation (DA) and deflection amplitude (DeflA), that have been described in previous studies [20, 31]. The latest major update released new keratoconus-associated DCR parameters and a biomechanical corrected intraocular pressure (bIOP) that is less influenced by age, central corneal thickness (CCT), and other DCR parameters [32]. As bIOP was developed for human eyes, it was excluded from further analysis because of the use of porcine eyes in our study. Further, maximal inverse radius (1/*R*) is the radius at highest concavity (HC-R) where the integrated radius defines the sum of the reciprocal curvature (1/*R*) between A1 and A2 [22]. Another parameter is DA ratio that illustrates the ratio of peripheral (at 1 mm and 2 mm) and central deformation [22]. Further, there is a stiffness parameter (SP A1) calculated at A1 which is described as the difference of adjusted external pressure and bIOP divided by A1 deflection amplitude [23].

Mean corneal stiffness (Kc[mean]) and constant corneal stiffness (Kc[linear]) were calculated from raw data of Corvis ST using a novel analytical model [29, 30]. Kc[mean] describes the non-linear elastic behavior, whereas Kc[linear] describes linear elastic behavior of the corneal tissue. The model was solved using non-linear least square technique MATLAB R2013a (MathWorks, Inc., USA).

### Statistical analysis

Statistical analysis was performed using Excel (2016, Microsoft Corp.) and SPSS (version 25, IBM Corp.). Normal distribution was confirmed by Shapiro-Wilk test and *Q*-*Q* plots. For multiple comparisons between all groups at one time level (baseline data, stress-strain data), one-way analysis of variance (ANOVA) with Bonferroni post hoc correction was used. The measurements were analyzed as repeated measurements that were evaluated by linear mixed models. Further, changes in corneal thickness before and after treatment were considered as covariate. A *P* value less than 0.05 was considered statistically significant.

## Results

### Stress-strain extensometry

According to the workflow, stress-strain measurements were performed after CXL treatment for groups 2, 3, and 4. The tissue of group 1 was soaked in riboflavin and did not obtain CXL. Therefore, it was considered as the control group. Corneal thickness, which was measured by ultrasound pachymetry in corneal strips before stress-strain measurement, showed significantly higher values in the control group compared to A-CXL (18*5) (*P* = 0.026), whereas no other significances were observed (all *P* > 0.05, Table 1).

**Table 1:** Descriptive analysis of corneal strips of porcine eyes concerning stress-strain measurement. Significance is marked in italics

	Controls (*n* = 18)	S-CXL (3*30) (*n* = 18)	A-CXL (9*10) (*n* = 18)	A-CXL (18*5) (*n* = 18)	*P* value^1^	*P* value^2^	*P* value^3^
Mean ± SD							
Length of sample [mm] by a preload of 0.02 N/m^2^	7.18 ± 0.2	7.02 ± 0.19	7.18 ± 0.35	7.28 ± 0.41	0.720	1.0	1.0
Corneal thickness [mm]	0.74 ± 0.09	0.69 ± 0.09	0.69 ± 0.07	0.67 ± 0.06	0.265	0.253	*0.026*

*A-CXL* accelerated CXL, *S-CXL* standard CXL

^1^*P* value between controls and S-CXL (3*30)

^2^*P* value between controls and A-CXL (9*10)

^3^*P* value between controls and A-CXL (18*5)

**Fig. 6:**
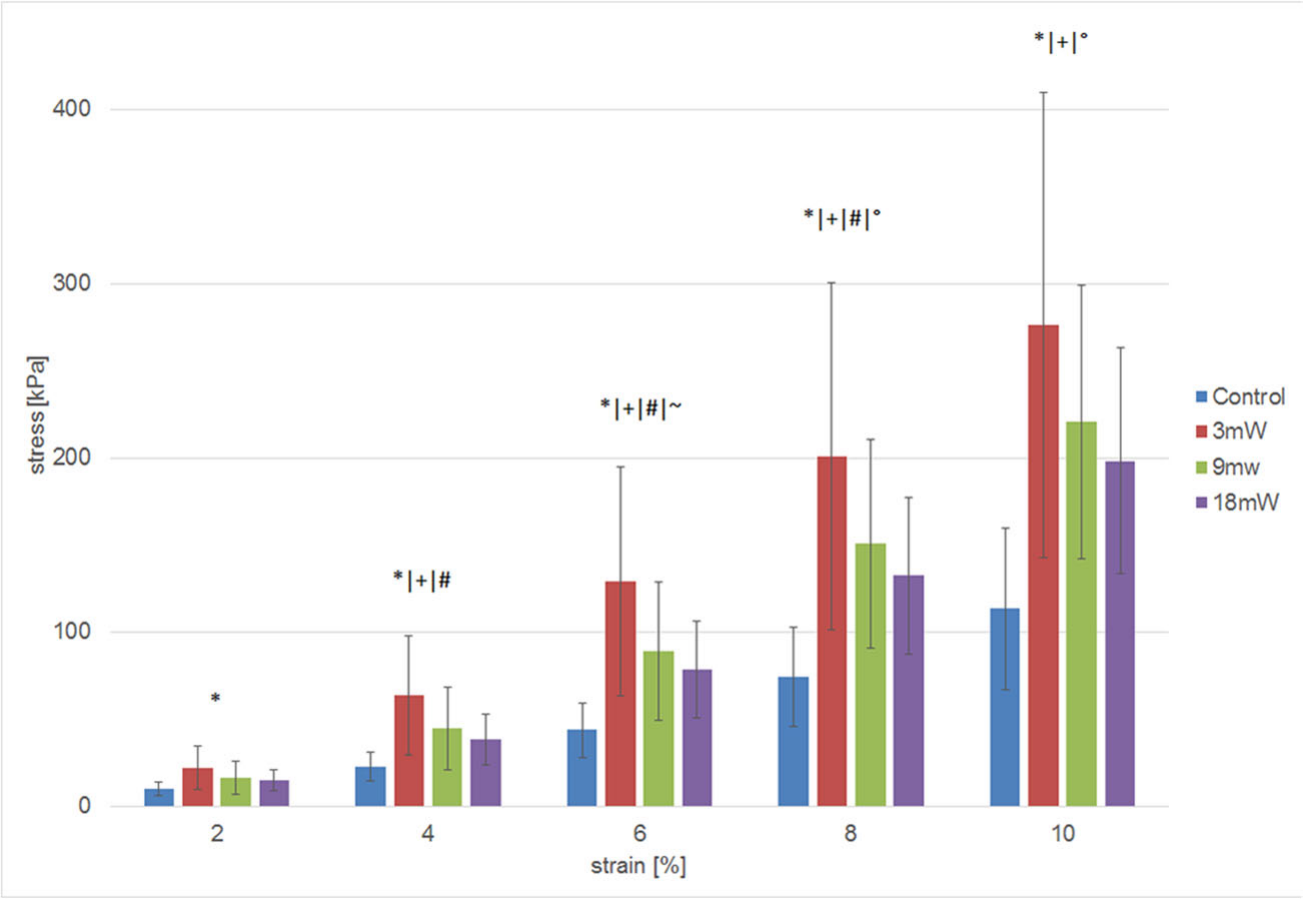
Stress-strain bar plot of extensometer measurements in different CXL protocols. *P < 0.05 between 3 mW and controls. ^+^*P* < 0.05 between 9 mW and controls. ^#^*P* < 0.05 between 3 mW and 18 mW. ^~^*P* < 0.05 between 3 mW and 9 mW. °*P* < 0.05 between 18 mW and controls

Pre-measurement length adjustment of corneal strips was 7 mm in all groups. There were no significant differences between the four groups, although A-CXL (18*5) had slightly longer clamped strips (all *P* > 0.05). The bar graph of stress-strain measurements (Fig. 6) showed that stress was significantly higher in S-CXL (3*30) compared to the controls in all parts of the strain (all *P* < 0.05). Also, stress was significantly higher for S-CXL (3*30) than A-CXL (18*5) at 4%, 6%, and 8% strain (all *P* < 0.05), but not at 2% and 10%. A significantly higher stress for S-CXL (3*30) compared to A-CXL (9*10) could only be observed at 6% strain (*P* < 0.05). For A-CXL (9*10), there was a significantly higher stress at 4%, 6%, 8%, and 10% strain compared to controls, whereas no increased stress was found in comparison to A-CXL (18*9). Further, stress was significantly higher for A-CXL (18*5) than the controls at 8% and 10% strain.

Tensile stiffness (K[ts]) was derived from the load to the extension plot by calculating the slope in defined points of extension. The mean extensions that correspond to a strain of 2%, 4%, 6%, 8%, and 10% were 0.15 mm, 0.29 mm, 0.44 mm, 0.58 mm, and 0.72 mm, respectively. The results are presented in Table 2. K[ts] was significantly higher for S-CXL (3*30) compared to controls (*P* < 0.05 in all points), A-CXL (9*10) (*P* < 0.05 between 0.15 and 0.44 mm), and A-CXL (18*5) (*P* < 0.05 between 0.15 and 0.44 mm). In longer extensions, A-CXL (9*10) and A-CXL (18*5) showed significantly higher K[ts] than controls. However, they did not differ from each other in all points (*P* = 1.0).

**Table 2:** Comparison of tensile stiffness (K[ts]) derived from load to extension plot. Significance is marked in italics

	*N*	K[ts] at 0.15 mm	K[ts] at 0.29 mm	K[ts] at 0.44 mm	K[ts] at 0.58 mm	K[ts] at 0.72 mm
Controls	18	231.2 ± 113.1	425.3 ± 198.7	650.6 ± 282.8	936.0 ± 428.6	1164.5 ± 533.3
S-CXL (3*30)	18	828.6 ± 512.8	1440.5 ± 875.6	1958.9 ± 1062.1	2135.3 ± 997.2	1975.9 ± 803.8
*P* value (S-CXL (3*30) − controls)		*< 0.001*	*< 0.001*	*< 0.001*	*< 0.001*	*0.03*
A-CXL (9*10)	18	472.7 ± 346.1	896.0 ± 403.7	1264.6 ± 574.9	1676.4 ± 624.8	1747.7 ± 637.3
*P* value (A-CXL (9*10) − controls)		0.204	0.052	*0.046*	*0.011*	0.062
*P* value (A-CXL (9*10) − S-CXL (3*30))		*0.013*	*0.016*	*0.017*	0.285	1.0
A-CXL (18*5)	18	489.2 ± 228.9	823.5 ± 352.2	1325.9 ± 512.2	1706.1 ± 539.4	1838.1 ± 650.0
*P* value (A-CXL (18*5) − controls)		0.143	0.152	*0.021*	*0.007*	*0.020*
*P* value (A-CXL (18*5) − S-CXL (3*30))		*0.020*	*0.004*	*0.037*	0.379	1.0
*P* value (A-CXL (18*5) − A-CXL (9*10))		1.0	1.0	1.0	1.0	1.0

*A-CXL* accelerated CXL, *CXL* corneal cross-linking, *K[ts]* tensile stiffness in (N/m), *S-CXL* standard CXL

### Corvis ST and corneal stiffness

The CXL groups did not differ in pre-treatment values for CVS-IOP, CVS-CT, SP A1, Kc[mean], and Kc[linear] (all *P* > 0.05, Table 3). The alterations in DCR parameters are presented in Table 4. For S-CXL (3*30), a highly significant increase was observed in CVS-IOP, DA ratio 2 mm, and SP A1 (all *P* < 0.05). A1 velocity, deflection amplitude, and integrated radius were decreased after treatment (all *P* < 0.05). Additionally, corneal thickness measured by Corvis ST (CVS-CT) was decreased (*P* = 0.001). Similar results were observed for A-CXL (9*10) and A-CXL (18*5), whereby DA ratio 2 mm did not change significantly for A-CXL (9*10) (all *P* = 0.062). By considering the reduction of CVS-CT during the procedure as covariates in linear mixed models, no effect on above-mentioned results was observed. Concerning corneal stiffness parameter Kc[mean] and Kc[linear], a significant increase was shown for all groups. Adjusting CVS-CT, Kc[mean] was not significantly increased for A-CXL (18*5). Comparing the pre- and post-treatment differences between the CXL protocols (Table 5), CVS-IOP increased significantly more in S-CXL (3*30) than in A-CXL (9*10) and A-CXL (18*5) as well as in A-CXL (9*10) rather than in A-CXL (18*5) (all *P* < 0.05). The increase after S-CXL(3*30) was significantly higher in DA ratio 2 mm, deflection amplitude, and SP A1 compared to either A-CXL (9*10) or A-CXL (18*5) or both (all *P* < 0.05). There was no statistical significance between the CXL groups concerning the pre- and post-treatment differences in integrated radius and Kc[mean] and Kc[linear] (*P* > 0.05).

**Table 3:** Descriptive analysis of pre-treatment DCR parameters between CXL groups

	S-CXL (3*30) (*n* = 18)	A-CXL (9*10) (*n* = 18)	A-CXL (18*5) (*n* = 18)	*P* value^1^	*P* value^2^	*P* value^3^
Mean ± SD						
CVS-IOP [mmHg]	9.89 ± 0.95	10.42 ± 0.93	10.11 ± 0.9	0.385	1.0	1.0
CVS-CT [μm]	699.7 ± 74.5	692.4 ± 58.8	697.7 ± 62.7	0.756	0.930	0.807
SP A1 [mmHg/mm]	75.46 ± 9.4	80.21 ± 9.08	76.61 ± 10.46	0.714	1.0	1.0
Kc[mean] [N/m]	78.08 ± 11.97	85.88 ± 10.74	82.93 ± 10.07	0.285	1.0	1.0
Kc[linear] [N/m]	81.58 ± 11.59	89.13 ± 8	84.16 ± 9.6	0.192	1.0	0.922

*A-CXL* accelerated CXL, *CVS-CT* corneal thickness measured by Corvis ST, *CVS-IOP* uncorrected IOP measured by Corvis ST, *CXL* corneal cross-linking, *IOP* intraocular pressure, *Kc* corneal stiffness derived from waveform analysis, *S-CXL* standard CXL, *SP A1* stiffness parameter at 1st applanation

^1^*P* value between S-CXL (3*30) and A-CXL (9*10)

^2^*P* value between S-CXL (3*30) and A-CXL (18*5)

^3^*P* value between A-CXL (9*10) and A-CXL (18*5)

**Table 4:** Comparison of different CXL protocols concerning DCR parameter outcome. Significance is marked in italics. All parameters were adjusted by corneal thickness (CT)

	S-CXL (3*30) (*n* = 18)			A-CXL (9*10) (*n* = 18)			A-CXL (18*5) (*n* = 18)		
	Pre-treatment	Post-treatment	*P* value	Pre-treatment	Post-treatment	*P* value	Pre-treatment	Post-treatment	*P* value
CVS-IOP [mmHg]	9.89 ± 0.95	12.861 ± 0.80	*< 0.001*	10.42 ± 0.93	12.44 ± 0.57	*< 0.001*	10.11 ± 0.90	11.25 ± 1.02	*< 0.001*
CVS-IOP [mmHg] adj. CT [estimated mean ± SE]	[9.98 ± 0.21]	[12.74 ± 0.21]	*< 0.001*	[10.48 ± 0.20]	[12.41 ± 0.203]	*< 0.001*	[10.19 ± 0.21]	[11.17 ± 0.21]	*< 0.001*
CVS-CT [μm]	699.72 ± 74.49	642.89 ± 49.85	*< 0.001*	692.39 ± 58.77	667.33 ± 47.79	*0.034*	697.67 ± 62.7	654.5 ± 61.71	*< 0.001*
A1 velocity [m/s]	0.16 ± 0.01	0.14 ± 0.01	*< 0.001*	0.15 ± 0.01	0.14 ± 0.01	*< 0.001*	0.16 ± 0.01	0.15 ± 0.01	*< 0.001*
A1 velocity [m/s] adj. CT [estimated mean ± SE]	[0.16 ± 0.0]	[0.14 ± 0.0]	*< 0.001*	[0.15 ± 0.0]	[0.14 ± 0.0]	*< 0.001*	[0.16 ± 0.0]	[0.15 ± 0.0]	*< 0.001*
Deflection Amp. [mm]	1.19 ± 0.1	1.02 ± 0.09	*< 0.001*	1.14 ± 0.07	1.01 ± 0.09	*< 0.001*	1.23 ± 0.12	1.15 ± 0.12	*< 0.001*
Deflection Amp. [mm] adj. CT [estimated mean ± SE]	[1.18 ± 0.02]	[1.02 ± 0.02]	*< 0.001*	[1.14 ± 0.02]	[1.01 ± 0.02]	*< 0.001*	[1.23 ± 0.02]	[1.15 ± 0.02]	*< 0.001*
DA ratio (2 mm)	3.97 ± 0.17	4.28 ± 0.22	*< 0.001*	3.9 ± 0.2	3.99 ± 0.27	0.062	3.85 ± 0.26	4.04 ± 0.27	*< 0.001*
DA ratio (2 mm) adj. CT [estimated mean ± SE]	[3.97 ± 0.06]	[4.28 ± 0.22]	*< 0.001*	[3.9 ± 0.06]	[3.99 ± 0.27]	0.075	[3.85 ± 0.06]	[4.04 ± 0.27]	*0.001*
Max. 1/*R* [mm^-1]	0.18 ± 0.01	0.17 ± 0.01	*< 0.001*	0.18 ± 0.01	0.17 ± 0.01	*< 0.001*	0.19 ± 0.01	0.18 ± 0.01	*< 0.001*
Max. 1/*R* [mm^-1] adj. CT [estimated mean ± SE]	[0.18 ± 0.0]	[0.17 ± 0.0]	*< 0.001*	[0.18 ± 0.0]	[0.17 ± 0.0]	*< 0.001*	[0.19 ± 0.0]	[0.18 ± 0.0]	*< 0.001*
Integrated radius [mm^-1]	9.87 ± 0.48	9.04 ± 0.47	*< 0.001*	9.36 ± 0.65	8.62 ± 0.47	*< 0.001*	10.03 ± 0.64	9.44 ± 0.78	*< 0.001*
Integrated radius [mm^-1] adj. CT [estimated mean ± SE]	[9.83 ± 0.14]	[9.09 ± 0.14]	*< 0.001*	[9.34 ± 0.14]	[8.63 ± 0.14]	*< 0.001*	[10 ± 0.14]	[9.47 ± 0.14]	*< 0.001*
SP A1 [mmHg/mm]	75.46 ± 9.4	100.13 ± 11.48	*< 0.001*	80.21 ± 9.08	98.02 ± 9.33	*< 0.001*	76.61 ± 10.46	84.69 ± 11.23	*0.001*
SP A1 [mmHg/mm] adj. CT [estimated mean ± SE]	[73.04 ± 1.95]	[103.45 ± 1.98]	*< 0.001*	[78.53 ± 1.94]	[98.87 ± 1.93]	*< 0.001*	[74.39 ± 1.95]	[86.84 ± 1.95]	*< 0.001*
Kc[mean] [N/m]	78.08 ± 11.97	91.54 ± 13.9	*< 0.001*	85.88 ± 10.74	99.58 ± 13.96	*< 0.001*	82.93 ± 10.07	89.64 ± 10.2	*0.043*
Kc[mean] adj. CT [estimated mean ± SE]	[78.13 ± 2.89]	[91.4 ± 2.98]	*< 0.001*	[85.91 ± 2.88]	[99.53 ± 2.9]	*< 0.001*	[82.98 ± 2.89]	[89.53 ± 2.93]	0.055
Kc[linear] [N/m]	81.58 ± 11.59	93.82 ± 13.11	*< 0.001*	89.13 ± 8	101.77 ± 10.04	*< 0.001*	84.16 ± 9.6	90.41 ± 9.41	*0.027*
Kc[linear] adj. CT [estimated mean ± SE]	[81.73 ± 2.53]	[93.44 ± 2.61]	*< 0.001*	[89.21 ± 2.52]	[101.62 ± 2.53]	*< 0.001*	[84.28 ± 2.53]	[90.14 ± 2.56]	*0.044*

*1/R* inverse radius, *A-CXL* accelerated CXL, *A1* applanation 1, *adj* adjusted, *Amp* amplitude, *CT* corneal thickness measured by Corvis ST, *CVS* Corvis ST, *CVS-IOP* uncorrected Corvis ST IOP, *CXL* corneal cross-linking, *IOP* intraocular pressure, *DA* deformation amplitude, *Kc* corneal stiffness derived from waveform analysis, *max* maximal, *S-CXL* standard CXL, *SE* standard error, *SP A1* stiffness parameter at 1st applanation

**Table 5:** Comparison of differences between pre- and post-treatment in performed CXL protocols. Significance is marked in italics

Parameter	Difference of S-CXL (3*30)	Difference of A-CXL (9*10)	Difference of A-CXL (18*5)	*P* value (3*30–9*10)	*P* value (3*30–18*5)	*P* value (9*10–18*5)
CVS-IOP [mmHg]	+ 2.97 ± 0.76	+ 2.03 ± 0.79	+ 1.14 ± 1.04	*0.006*	*0.000*	*0.011*
CVS-CT [μm]	− 56.83 ± 55.13	− 25.06 ± 58.8	− 43.17 ± 25.43	0.169	1.000	0.812
A1 velocity [m/s]	− 0.02 ± 0.01	− 0.01 ± 0.01	− 0.01 ± 0.01	0.973	0.258	1.000
Deflection Amp [mm]	− 0.17 ± 0.04	− 0.14 ± 0.05	− 0.09 ± 0.09	0.575	*0.001*	*0.042*
DA ratio 2 mm [mm]	+ 0.31 ± 0.16	+ 0.09 ± 0.23	+ 0.19 ± 0.23	*0.009*	0.287	0.496
Integrated radius [mm^-1]	− 0.83 ± 0.36	− 0.74 ± 0.36	− 0.59 ± 0.55	1.000	0.344	0.912
SP A1 [mmHg/mm]	+ 24.66 ± 9.27	+ 17.81 ± 10.07	+ 8.08 ± 8.78	0.100	*0.000*	*0.009*
Kc[mean] [N/m]	+ 13.46 ± 18.63	+ 13.7 ± 8.64	+ 6.7 ± 11.42	1.000	0.423	0.383
Kc[linear] [N/m]	+ 12.23 ± 15.83	+ 12.64 ± 6.45	+ 6.26 ± 10.25	1.000	0.376	0.306

*A-CXL* accelerated CXL, *A1* applanation 1, *adj* adjusted, *Amp* amplitude, *CT* corneal thickness measured by Corvis ST, *CVS* Corvis ST, *CVS-IOP* uncorrected Corvis ST IOP, *CXL* corneal cross-linking, *IOP* intraocular pressure, *DA* deformation amplitude, *Kc* corneal stiffness derived from waveform analysis, *S-CXL* standard CXL, *SE* standard error, *SP A1* stiffness parameter at 1st applanation

## Discussion

Corneal cross-linking using riboflavin and ultraviolet-A light is an established treatment method to halt progression in keratoconus. It is defined as a photochemical process causing cross-links in corneal tissue, which proceeds mainly intra- and intermolecularly at the fibrils’ surface and within the cores of proteoglycan, in the area between the fibrils [33]. Several experimental studies confirmed the stiffening effects of corneal tissue [1, 8, 11, 34]. In vivo, the efficacy of CXL is detected by stabilization or flattening of keratometry values of corneal topography in short-term and long-term results [6, 35, 36]. Until now, an assessment of changes in biomechanical properties in vivo, after CXL, could only be performed by waveform-derived parameters (peak 1 and peak 2) using ORA [16, 37, 38]. Sedaghat and co-workers have shown alterations in DCR parameters after CXL, whereas no changes were observed for CH and CRF [27]. Further, Vinciguerra and co-workers have shown significant alterations in DCR parameters by the Corvis ST in preliminary results [26].

The aim of this study was to evaluate alterations in corneal biomechanical properties after application of CXL in porcine eyes using stress-strain extensometry and air-puff tonometry. This study has compared three CXL protocols, which are used in clinical practice, S-CXL (3*30), A-CXL (9*10), and A-CXL (18*5). The modification of intensity and time is according the Bunsen-Roscoe Law, while the total energy remains constant (5.4 J/cm^2^). According to experimental methods of this study, all specimens were soaked in riboflavin solution containing 10% dextran to avoid corneal swelling. It is known that biomechanical properties of the cornea depend on corneal hydration, whereas hydrated corneas have weaker tensile properties than dehydrated corneas [39]. In case of CXL, Hatami-Marbini and Jayaram have shown that corneal stiffening depended on pre-treatment corneal hydration as well; however, the improvement of tensile properties after CXL was not different, if mechanical tests were done under the same hydration conditions [40]. Further, riboflavin with dextran tends to decrease corneal thickness during CXL treatment [41]. Therefore, we have decided to use a dextran concentration of 10%. Nonetheless, corneal thickness alterations were considered for stress-strain as well as Corvis ST measurements.

In our study, we found an increased stiffness for S-CXL (3*30), A-CXL (9*10), and A-CXL (18*5) at 10% strain compared to controls. These results are similar to investigations of Schumacher et al., Krueger et al., and Wernli et al. The latter found significant stiffening up to 45 mW/cm^2^ [8]. In contrast, Hammer et al. showed that A-CXL (18*5) were not significantly stiffer than controls [11]. The reason for the different outcomes might be various experimental set-ups that are summarized in Table 6. Additionally, inflation tests performed by Bao et al. have shown that the shorter the irradiation time is, the lower is the increase in stiffening the tissue [42]. However, these results were observed at 4% strain that is equal approximately to physiological strain of the cornea under normal IOP conditions [42]. Our results confirm these findings with regard to the stress-strain plot. At 2% strain, corneas treated with S-CXL (3*30) were significant stiffer than the controls. At 4% strain, both S-CXL (3*30) and A-CXL (9*10) were stiffer than the controls, whereas S-CXL (3*30) was stiffer than A-CXL (18*5). Nevertheless, it can be assumed that accelerated protocols are effective up to 9 mW/cm^2^ with a total intensity of 5.4 J/cm^2^. Further, tensile stiffness (K[ts]) was calculated from load to extension plots and showed similar results as in stress-strain. However, K[ts] was not significantly higher for A-CXL (9*10) in comparison to controls at 0.72 mm (equal to 10% strain), whereas a significant stiffening occurred at 0.58 mm (equal to 8% strain).

**Table 6:** Comparison of experimental ex vivo investigations concerning induced corneal stiffness by cross-linking with riboflavin solution and ultraviolet-A irradiation in porcine eyes

Group	Specimen	Irradiation area	Test and sample dimensions	UV intensity	Chromophore (centration)
Schumacher et al. [51]	Porcine	9 mm	Uniaxial, 7 mm × 1 mm	3 mW and 9 mW	0.1% riboflavin with 20% dextran
Krüger et al. [52]	Porcine	n.a.	Uniaxial, 8 mm × 5 mm	2 mW to 15 mW	0.1% riboflavin with CMC
Wernli et al. [8]	Porcine	~ 9 mm	Uniaxial, 7 mm × 5 mm	3 mW to 90 mW	0.1% riboflavin
Hammer et al. [11]	Porcine	11.3 mm	Uniaxial, 10 mm × 5 mm	3 mW to 15 mW	0.1% riboflavin
Kling et al. [34]	Porcine	n.a.	2-dimensional, 10 mm	1.5 mW and 3 mW	0.1% riboflavin
Bao et al. [42]	Japanese rabbits	9 mm	Inflation tests	3 mW to 90 mW	0.22% riboflavin

*CMC* carboxymethylcellulose, *n.a.* not applicable

In this study, we examined the differences between the pre- and post-treatment of certain DCR and stiffness parameters (Kc[mean, Kc[linear]) using the complete porcine eye globe that was mounted in a special silicon case under a water-column-controlled intraocular pressure of 20 mmHg. This means that our experimental set-up took into account effects of ocular tissue (e.g., sclera) of the whole eye globe [43]. The changes in DCR parameters and Kc[mean/linear] confirmed the stiffening effect of CXL in all used protocols. First, we observed a significant decrease of corneal thickness (CT) measured by Corvis before and after CXL. Thus, each parameter was separately analyzed, considering the impact of CT. In all three CXL protocols, the CVS-IOP was measured to be significantly higher after treatment, while the IOP was adjusted to a constant at 20 mmHg over the whole procedure. This indicates that elevated measured IOP after CXL showed a stiffer behavior of the eye wall, especially the cornea. An increased IOP in Goldmann applanation tonometry after CXL was reported in vivo by Kymionis et al. and Kasumovic et al. [44, 45]. The deflection amplitude was significantly decreased in all protocols, even though CT was adjusted. This leads to the assumption that the cornea is less deformable against the air puff. Further, the significant reduction of maximal inverse radius (1/*R*) indicates a flattened concave radius at highest concavity (HC-R) in all protocols. In vivo, Sedaghat et al. and Hashemi et al. have also observed an increased HC-R after CXL in standard [27] and accelerated protocols [46], respectively. Following this, values of integrated (inverse) radius were significantly decreased in our results for all protocols. This was also shown in vivo for standard [27] and accelerated protocols [26, 47, 48]. Additionally, in recent studies, the deformation amplitude ratio (DA ratio) was decreased after CXL in different cohorts, whereby not all data showed significance [26, 27, 47, 48]. These results are in line with the definition, which states that higher values of the DA ratio are associated with softer corneas [22]. Therefore, a decrease of the DA ratio is expected after CXL. In contrast, our experimental results showed that the DA ratio (2 mm) increases in all protocols after CXL, even though the deformation amplitude (data not shown) and deflection amplitude were reduced. Coming back to its definition, this might explain that CXL led to less deformation of the peripheral cornea and, thus, a higher DA ratio. On the other hand, the higher thickness and the “normal” peripheral thickness profile of the porcine eye model possibly distorted these results.

SP A1 is the stiffness parameter calculated for reaching the first applanation (A1), which was significantly increased in standard and accelerated protocols. In literature, there are some in vivo reports that have shown increased values as well but not all were significant [26, 27, 47, 48]. Corneal stiffness expressed in newtons per meter (Kc[mean], Kc[linear]) showed a similar increase in S-CXL and A-CXL (9*10), whereas A-CXL (18*5) was less pronounced compared to SP A1.

Stress-strain extensometry confirmed stiffening of the corneal tissue for S-CXL (3*30) and A-CXL (9*10) in physiological conditions (at 4% strain) and for all CXL protocols at 10% strain. Previously, the same specimens were examined with the Corvis ST to obtain alterations before and after CXL in DCR parameters. Therefore, it can be assumed that the corneal stiffening induced by the CXL procedure has affected the biomechanical properties of the cornea. Stiffness is described as the resistance of an object against a deformation [23]. A higher stiffness of the cornea implicates a less deformation to an applied force to the cornea. Hence, a greater force is necessary to deform the cornea, e.g., in IOP measurements, whereby the IOP is overestimated [23]. In our study, we have applied a constant IOP to the eye globe during the whole procedure. Our results confirmed corneal stiffening by overestimation of CVS-IOP after CXL treatment under unchanged environmental conditions. Further, velocity until 1st applanation and deflection amplitude was lower after CXL due to higher stiffness and greater resistance against the air puff. However, deflection amplitude and CVS-IOP have a strong relationship and should be used with caution as single parameter to evaluate alterations in biomechanical properties after CXL in clinical practice [26, 32]. Also, the shape of the inward movement of the stiffened cornea has been altered; thus far, the maximal 1/*R* was reduced implying a flatter curvature at HC [23, 26]. Therefore, the integrated inverse radius has been reduced as the cornea became stiffer. SP A1, Kc[mean], and Kc[linear] are parameters that describe the resistance against deformation (at 1st applanation) and non-linear as well as linear behavior based on the deformation amplitude of the corneal tissue, respectively. These parameters were observed to be lower in softer eyes like keratoconus [23, 29]. In our study, they showed higher values after CXL implicating stiffening of the corneal tissue. None of the other DCR parameters were analyzed due to their low importance in clinical practice. Further, DCR parameters that describe the outward movement until 2nd applanation are influenced by viscoelastic properties of the tissue and are not comparable to uniaxial strip extensometry.

Comparing the differences after treatment in all protocols, there is a trend that S-CXL induced a higher change in corneal response to the air puff, than A-CXL protocols. However, significance was only shown in CVS-IOP. Additionally, a higher stiffening effect of S-CXL than A-CXL (18*5) was seen in the deflection amplitude and SP A1. Further, the increase of Kc[mean] and Kc[linear] is more pronounced in S-CXL than in A-CXL protocols, whereby no significance was achieved.

The study is limited in the way that only ex vivo porcine eyes were used to evaluate CXL effects with stress-strain measurements and Corvis ST. Previous studies have shown that porcine eyes are less stiff than human donor eyes, measured in corneal [49] and scleral tissue [50]. Another limitation is that it was not possible to use paired eyes of the same pig to compare different CXL protocols.

In conclusion, in this study, we showed that Corvis ST is able to detect effects of corneal cross-linking in the biomechanical behavior of ex vivo porcine eyes, under laboratory conditions. Furthermore, this study investigated commonly used CXL protocols that increase the corneal stiffness, provably by stress-strain measurements. Thus, changes in DCR parameters captured by Corvis ST were validated by stress-strain measurements. Corvis ST might be useful to assess effectiveness of CXL and support topographic and tomographic data during follow-up examinations.
